# Effects of school-based physical activity programs on executive function development in children: a systematic review

**DOI:** 10.3389/fpsyg.2025.1658101

**Published:** 2025-09-03

**Authors:** Javier González-del-Castillo, Irene Barbero-Alcocer

**Affiliations:** ^1^Department of Sports Sciences, Faculty of Medicine, Health and Sports, Universidad Europea de Madrid, Madrid, Spain; ^2^Department of Education and Teacher Training, Faculty of Law, Education and Humanities, Universidad Europea de Madrid, Madrid, Spain

**Keywords:** executive functions, physical activity interventions, cognitive development, school-aged children, neuroplasticity, inhibitory control, working memory, cognitively engaging exercise

## Abstract

**Background:**

Executive functions (EF) are core cognitive processes that support self-regulation, learning, and behavioral flexibility in childhood. Structured physical activity (PA) programs implemented in school settings have been proposed as a means to enhance EF, but previous findings are inconsistent due to variations in intervention design, cognitive demands, and measurement strategies. This review offers an updated synthesis by focusing exclusively on school-based interventions in primary school children and including studies with neurophysiological outcomes.

**Methods:**

A systematic search was conducted in PubMed, SCOPUS, Web of Science, and EBSCO for studies published between January 2015 and March 2025. Eligible studies were randomized or cluster-randomized trials evaluating structured PA programs targeting EF in children aged 6–12 years. Methodological quality was assessed using a custom checklist aligned with Cochrane ROB-2 criteria. Due to heterogeneity in intervention formats and outcome measures, a narrative synthesis was conducted.

**Results:**

Ten studies met the inclusion criteria (total *N* ≈ 2,400). Short, cognitively engaging exercise sessions, such as rhythm-based activities or task-switching drills, were frequently associated with immediate improvements in inhibitory control. Longer-term interventions delivered over several weeks showed more robust and consistent benefits, particularly for inhibitory control and working memory. Positive effects were reported in 6 of 8 studies assessing inhibition, 5 of 6 on working memory, and 3 of 4 on cognitive flexibility. Some studies using fNIRS and EEG reported changes in prefrontal activation, suggesting potential functional enhancement. However, overall methodological quality was moderate, with common limitations in blinding and protocol transparency.

**Conclusion:**

School-based physical activity can support EF development in children, especially when interventions are sustained and cognitively demanding. Effects are strongest for inhibition and working memory, while gains in cognitive flexibility appear less consistent and may require greater novelty and task variability. Future trials should refine intervention parameters, apply standardized EF assessments, and explore individual variability to guide evidence-based educational applications.

**Systematic review registration:**

CRD420251084225, https://www.crd.york.ac.uk/PROSPERO/view/CRD420251084225.

## 1 Introduction

### 1.1 Rationale and current relevance of the study

Executive functions (EF) are higher-order cognitive processes essential for behavior regulation, learning, and social development throughout childhood. Core components, working memory, inhibitory control, and cognitive flexibility, enable children to manage tasks, shift attention, and adapt to changing demands with cognitive control and flexibility (Miyake et al., 2000; Diamond and Ling, 2016). These skills can be reliably assessed using standardized batteries such as the NIH Toolbox (Zelazo et al., 2013) and form the foundation for academic success and adaptive functioning (Diamond, 2013; Zelazo et al., 2008).

A strong development of EF in early years has been consistently linked to better academic performance, emotional regulation, and social competence. For example, Blair and Razza (2007) showed associations with foundational literacy and math skills, while Blair and Diamond (2008) emphasized the role of EF in self-regulation. Longitudinal studies suggest that early executive-related abilities predict school readiness and later achievement (Duncan et al., 2007; McClelland et al., 2007), and that preschool levels of cognitive control forecast EF performance well into adolescence (Eigsti et al., 2006). These findings support a developmental continuity in executive functioning (Barkley, 2012; Diamond, 2013).

In contrast, deficits in EF are associated with persistent learning difficulties, impulsivity, and behavioral problems. They can also contribute to significant impairments in daily functioning and occupational outcomes in later life (Barkley and Fischer, 2011; Barkley and Murphy, 2010; Gilbert and Burgess, 2008).

Recent studies have highlighted the potential of physical activity (PA) to enhance EF in school-age populations. Evidence suggests that exercise programs with cognitively demanding elements, such as those involving planning, attention, or rapid decision-making, can improve EF by stimulating neuroplastic changes in brain regions linked to cognitive control, particularly the dorsolateral prefrontal cortex (Diamond and Ling, 2016; Mao et al., 2024). Some of these changes have been associated with elevated levels of brain-derived neurotrophic factor (BDNF), a key mediator of synaptic growth and neural connectivity (Diamond, 2013; Kamijo et al., 2011). Aerobic exercise, in particular, has been shown to increase cerebral blood flow and BDNF concentrations in the prefrontal cortex, reinforcing the neurophysiological basis for EF improvement (Colcombe et al., 2006).

The setting where PA is implemented plays a critical role. School-based programs offer structure, reach a broad and diverse population, and allow integration into daily routines with relatively high adherence (Álvarez-Bueno et al., 2017; Watson et al., 2017). Interventions that balance moderate physical intensity with cognitive and emotional engagement tend to be more effective than those focused solely on movement or fitness (Vazou et al., 2019).

Despite growing interest, research in this area still faces important challenges. Studies vary widely in design, intervention type, and outcome measures (Dixon et al., 2025; Watson et al., 2017). Mao et al. (2024) point to inconsistencies in the duration, frequency, and structure of the programs, which make it difficult to identify what works best and under what conditions. Diamond and Ling (2016) note that cognitive benefits are uneven across PA types. Activities that embed executive demands within movement appear to yield stronger outcomes, particularly in working memory and inhibitory control.

This review addresses the need to examine the role of structured PA in supporting EF development during primary school years. The aim is to provide a clear and evidence-based synthesis of the most recent findings to guide educational practices and inform policy decisions.

Although this review follows the tripartite model of executive functions proposed by Miyake et al. (2000), inhibitory control, working memory, and cognitive flexibility, it is important to recognize that this approach reflects a predominantly cognitive view. Broader theoretical frameworks offer alternative ways of understanding executive functioning. Barkley (2012) emphasizes emotional and motivational self-regulation and the ability to manage behavior over time. Zelazo et al. (2008) propose a hierarchical model of EF development that includes emotional and social cognition. Blair and Raver (2015) advocate for an ecologically grounded model, where executive functioning emerges from the interaction between neurobiological systems and environmental stressors. These perspectives offer richer conceptualizations of EF, but they pose challenges for operationalization and comparability across empirical studies. Given the aims of this review, to compare measurable outcomes from structured interventions in school settings, a more pragmatic and standardized model was adopted. This decision allows for systematic synthesis while acknowledging the limitations of focusing primarily on the cognitive dimensions of EF.

### 1.2 Definition and development of the executive function concept

Executive functions (EF) refer to a set of higher-order cognitive abilities that support the regulation of thought, behavioral inhibition, goal planning and flexible adaptation to changing environments. Diamond (2013) emphasizes their importance in achieving deliberate goals and fostering intellectual autonomy from early childhood. The three core components are working memory, inhibitory control and cognitive flexibility. These elements interact to support self-regulated, goal-directed behavior.

One of the most widely cited frameworks in empirical research is that of Miyake et al. (2000), who identified these three components as distinct yet interrelated factors using latent-variable analyses. This model has proven influential in applied settings, particularly in education and clinical intervention, as it offers a practical structure for assessment and developmental monitoring. In a complementary view, Baddeley (1986) underscored the central role of working memory in coordinating mental activity, especially during complex learning tasks.

Still, this perspective has limitations. Several authors have proposed broader models that include emotional, motivational and contextual dimensions. Barkley (2012), for instance, argues that EF should not be seen solely as cold cognitive processes, but as mechanisms of self-regulation rooted in time management, emotional control and motivation. His model incorporates constructs such as retrospective foresight, proactive behavior and sustained effort, which are especially relevant when explaining children’s functioning in real-life environments.

A developmental approach has been suggested by Zelazo et al. (2008), who define EF as a set of hierarchically organized processes that gradually evolve throughout childhood. These range from basic forms of control to more reflective and deliberate self-regulation. This conceptualization has influenced tools such as the NIH Toolbox, which recognizes that executive growth involves not just acquiring isolated skills but progressively integrating complex regulatory processes.

Although this review adopts Miyake’s model as a guiding reference, primarily for its conceptual clarity and strong empirical foundation, it acknowledges that this framework offers a simplified but useful representation. Focusing on the three core components enables reliable measurement of intervention outcomes but does not capture the full functional complexity of executive systems. Current literature emphasizes that EF emerges in interaction with real-world environments, shaped by affective states, social demands and contextual variability (Banich, 2009; Blair and Raver, 2015).

This review builds on an operational model that is widely validated while remaining open to theoretical contributions that help interpret findings from a more ecologically grounded and multidimensional perspective on executive functioning.

### 1.3 Theoretical and empirical evidence on the effects of physical activity on executive functions

The relationship between physical activity (PA) and executive functions (EF) has been extensively explored from neuroscientific, cognitive and educational perspectives. Over the past two decades, empirical research has provided consistent evidence that PA can lead to measurable improvements in EF, especially when the interventions are structured, sustained and cognitively engaging (Diamond and Lee, 2011; Tomporowski et al., 2011).

From a physiological standpoint, regular PA increases the concentration of neurotrophic factors such as BDNF, IGF-1 and VEGF, which support neurogenesis, synaptogenesis and neural plasticity (Kamijo et al., 2011). These neurobiological changes typically occur in brain regions associated with executive control, including the prefrontal cortex, hippocampus and attentional systems. Hillman et al. (2008) observed that children and adolescents who participate in regular PA tend to have greater gray matter volume in areas involved in self-regulation and cognitive flexibility. Similarly, Colcombe et al. (2006) demonstrated that aerobic exercise enhances prefrontal cortical activation and gray matter density in adults, suggesting similar mechanisms may be active in children.

Davis et al. (2011) provided further support through a randomized controlled trial showing that a moderate-intensity aerobic program improved both EF performance and prefrontal cortex activation in overweight children, as measured through functional MRI. Their findings point to a causal link between physical training and neural enhancement, confirming that these changes are not merely behavioral correlates but reflect deeper neurophysiological adaptation.

Still, not all forms of physical activity produce the same cognitive outcomes. Diamond and Ling (2016) argue that activities involving only repetition or continuous exertion, such as running in a straight line, tend to show limited cognitive impact. In contrast, tasks that involve problem-solving, inhibition, rule changes or social interaction have stronger effects. These include team sports, cooperative games and rhythmic motor activities with embedded cognitive demands.

Several studies support this claim. Schmidt et al., (2016) found that active classroom breaks combining motor and cognitive elements improved children’s attention and working memory, while passive or purely physical breaks did not yield the same benefits. Pesce et al. (2009) also reported that even brief coordinative activities improved immediate recall, reinforcing the idea that the cognitive quality of the activity is as important as its duration or intensity.

It is also important to distinguish between acute and chronic effects. Chang et al. (2012) showed that a single bout of aerobic exercise can generate short-term improvements in attention and processing speed, mediated by arousal and neuromodulatory changes. Dixon et al. (2025) reached similar conclusions in their review of brief interventions, suggesting that short PA sessions may be strategically useful before cognitively demanding tasks. However, more durable effects require repeated practice. Mao et al. (2024) found that programs lasting at least 4 weeks were more likely to improve working memory and inhibitory control in a sustained manner.

In summary, physical activity has considerable potential to support executive function development in childhood, but its effectiveness depends on several interrelated variables. These include the cognitive complexity of the activity, the consistency and duration of the intervention and the developmental characteristics of the child population involved. This review builds on that evidence to clarify the conditions under which PA can most effectively enhance executive functioning during the primary school years.

To enhance conceptual clarity and support interpretation of the findings, this review adopts operational definitions for several frequently used terms. Cognitive engagement refers to the activation of mental processes such as attention, reasoning, or decision-making during physical activity. When these demands specifically target core executive functions, namely inhibitory control, working memory, and cognitive flexibility, they are referred to as executive demands. The term deliberate play describes structured yet flexible activities that promote exploration, problem-solving, and autonomy without rigid instruction or performance pressure. Coordinative complexity, by contrast, relates to the motor difficulty of a task, including elements such as dynamic balance, rhythmic sequencing, or bilateral integration. While these constructs often overlap, they are not interchangeable. For example, a physically complex task may not involve significant executive demands, whereas an activity that combines motor coordination with rule changes or planning requirements may engage both systems simultaneously.

### 1.4 Why focus on school-based programs

Schools offer a unique context for interventions aimed at strengthening executive functions (EF). Their universal access, structured routines and integration into children’s daily lives make them especially suitable for delivering physical activity (PA) programs. From both a practical and equity-based perspective, the school environment is one of the few settings where broad populations can be reached systematically, including children from diverse socioeconomic backgrounds under relatively consistent conditions (Watson et al., 2017; Álvarez-Bueno et al., 2017).

Empirical reviews have shown that school-based PA interventions benefit not only physical health but also key cognitive functions such as working memory, sustained attention and inhibitory control. Álvarez-Bueno et al. (2017), in a comprehensive meta-analysis, reported that structured school programs have a significant impact on overall academic performance and on specific EF outcomes. When these interventions are embedded in regular class time and aligned with educational objectives, they tend to show higher levels of adherence and feasibility.

The school setting also allows for more precise monitoring of intervention fidelity and for the use of standardized outcome measures. It provides a natural environment where cognitive demands, behavioral expectations and social interactions are already part of the daily routine. This enhances the ecological validity of EF practice and reinforces the transfer of skills to real-world settings (Diamond and Ling, 2016; Vazou et al., 2019).

Several initiatives have shown how integrating EF-relevant physical activity into classroom practice can yield sustained cognitive benefits. One notable example is the Tools of the Mind program, originally designed for preschool education and later adapted to early primary grades. It incorporates guided pretend play, advance planning and motor self-regulation as part of the daily curriculum (Diamond et al., 2007). Another study by Lakes and Hoyt (2004) found that school-based martial arts practice improved self-regulation and inhibitory control in young students, without requiring modifications to out-of-school routines.

The appeal of school-based programs extends beyond their cognitive outcomes. As Davis et al. (2011) note, integrating physical activity into the school day also contributes to the prevention of metabolic and emotional risk factors, making it a strategic component of public health efforts. International education and health agencies increasingly recognize the value of such interventions, supporting the integration of movement as a fundamental feature of the school experience rather than a supplementary activity.

Compared to extracurricular or pilot-based initiatives, school-based programs offer a compelling combination of effectiveness, reach and long-term sustainability. They are well-positioned to promote executive functioning in a way that is scalable, evidence-based and aligned with the broader goals of inclusive and holistic education.

### 1.5 Limitations of previous reviews and contributions of the present study

Although research on the role of physical activity (PA) in the development of executive functions (EF) has expanded in recent years, many existing reviews still face important limitations that weaken the strength and applicability of their conclusions in educational contexts. A common issue is the inclusion of highly heterogeneous samples in terms of age, educational stage and intervention settings. Reviews often group together studies involving preschool children, adolescents and young adults, making it difficult to identify patterns specific to middle childhood (Watson et al., 2017; Álvarez-Bueno et al., 2017).

Methodological inconsistencies also pose a challenge. Mao et al. (2024) note that many prior reviews lack precise reporting on the intensity, frequency and cognitive complexity of the interventions analyzed, limiting the ability to assess their true impact on different EF components. Dixon et al. (2025) highlight the wide variation in outcome measures, ranging from standardized cognitive tests to subjective reports, which introduces bias and reduces comparability across studies. Reloba et al. (2016) had already drawn attention to this problem nearly a decade earlier, emphasizing the need for uniform criteria in measuring executive outcomes.

Another significant shortcoming is the absence of a coherent theoretical framework for classifying and interpreting interventions. Banich (2009) observed that many reviews focus on superficial associations between physical activity and cognitive outcomes without explaining the mechanisms behind them. Without a neuropsychological model linking types of PA to specific executive systems, it is difficult to determine which activities are effective, and why. As a result, many findings remain limited to general statements about academic improvement, with little insight into the cognitive processes involved.

Furthermore, the integration of neurophysiological and neuroimaging data remains rare. Although some studies have incorporated tools such as functional MRI or event-related potentials, these are still exceptions rather than the rule (Rueda et al., 2005; Davis et al., 2011). The scarcity of neural evidence narrows our understanding of the brain-based mechanisms through which PA affects EF.

This review addresses these limitations through a more targeted and methodologically rigorous approach. It focuses specifically on children between the ages of 6 and 12, a developmentally coherent group that corresponds to the primary school years, where executive control develops most rapidly. It also includes only studies published between 2015 and 2025, a period marked by methodological improvements in trial design, intervention fidelity, and outcome measurement (Page et al., 2021).

Rather than merely asking whether PA enhances EF, this review seeks to clarify under what circumstances such benefits are most likely to emerge. It considers factors such as intervention duration, the presence of cognitive challenges, group dynamics and the type of instruments used to measure outcomes. In addition, it deliberately includes a subset of studies that incorporate neurophysiological and neuroimaging techniques, aiming to deepen our understanding of the mechanisms involved.

Taken together, these decisions reflect a commitment to advancing beyond previous reviews that were often limited by methodological heterogeneity, broad age ranges, and superficial interpretations. For example, Álvarez-Bueno et al. (2017) included studies spanning from preschool to adolescence and combined school-based and extracurricular interventions, which hindered the identification of consistent developmental patterns. Similarly, Singh et al. (2019) adopted a narrative approach without systematic appraisal of study quality or differentiation by intervention type. In contrast, the present review restricts the age range to 6–12 years, focuses exclusively on school-based interventions, and adheres strictly to PRISMA-P Group et al. (2015) guidelines. It incorporates detailed PICO tables, standardized flow diagrams, and structured risk of bias assessments. Furthermore, it considers moderators such as intervention duration, cognitive engagement, and the use of neurophysiological measures, offering a more precise and actionable synthesis. This approach seeks not only to summarize existing evidence but also to provide a conceptually grounded and practically relevant framework to guide the design of future school-based programs targeting executive function development.

### 1.6 Objectives and time frame of the review

The objective of this systematic review is to provide a rigorous synthesis of empirical evidence on the effects of physical activity (PA) programs implemented in school settings on the development of executive functions (EF) in children aged 6 to 12. The analysis focuses on both overall EF development and specific components, working memory, inhibitory control, and cognitive flexibility, while also examining intervention characteristics, assessment methods, and implementation conditions. This approach aligns with research indicating that in early and middle childhood, executive components often operate as a unified construct rather than as fully differentiated processes (Wiebe et al., 2008).

The decision to target the primary school age group responds to methodological and practical considerations. Limiting the age range reduces developmental variability across samples, increasing the comparability of results. At the same time, focusing on school-based interventions enhances ecological validity and potential for scalability in educational and public health contexts (Watson et al., 2017; Álvarez-Bueno et al., 2017).

The time frame selected (January 2015–March 2025) reflects a period of increased methodological refinement in the field. The widespread adoption of frameworks such as PRISMA-P Group et al. (2015), Page et al. (2021), the growth of randomized controlled trials (RCTs), and the integration of neurophysiological tools (e.g., fNIRS, EEG) have all contributed to more robust and nuanced evidence (Mao et al., 2024; Dixon et al., 2025).

This review builds on prior efforts by applying stricter inclusion criteria and emphasizing the interplay between intervention design and cognitive outcomes. It aims to fill existing gaps by offering a reliable foundation for future studies and informing evidence-based educational practices. The protocol was prospectively registered in the International Prospective Register of Systematic Reviews (PROSPERO; Registration ID: CRD420251084225).

## 2 Methodology

This review followed the Preferred Reporting Items for Systematic Reviews and Meta-Analyses (PRISMA) guidelines (PRISMA-P Group et al., 2015) and the recommendations described in the Cochrane Handbook for Systematic Reviews of Interventions (Higgins et al., 2024).

A systematic search was carried out in PubMed, SCOPUS, Web of Science, and EBSCO databases (including Academic Search Ultimate, APA PsycInfo, SPORTDiscus, MEDLINE Complete, Psychology and Behavioral Sciences Collection, ERIC, and Rehabilitation & Sports Medicine Source). The search covered studies published between January 2015 and March 2025 in peer-reviewed journals. The starting date was selected to capture recent studies reflecting advances in both exercise-cognition research and school-based implementation frameworks, following a surge in publications after 2015. The objective was to identify research examining the effects of physical activity interventions on executive functions in children aged 6 to 12 years.

The strategy combined controlled vocabulary terms (MeSH) and free-text keywords related to four categories: executive functions (e.g., “executive function*,” “working memory,” “inhibitory control,” “cognitive flexibility”), physical activity (e.g., “physical activity,” “exercise,” “sport,” “aerobic training”), intervention outcomes (e.g., “effect*,” “impact*,” “intervention*,” “influence”), and target population (e.g., “child,” “children,” “primary school,” “elementary school”). Detailed search strings for each database are provided in Supplementary material 1.

### 2.1 Eligibility criteria

The search was restricted to articles published in English. This review was limited to English-language publications due to resource constraints that precluded systematic translation. Eligibility criteria were defined using the PICOS framework (Population, Intervention, Comparison, Outcomes, and Study Design). Eligible studies included participants who were healthy children aged 6 to 12 years or attending primary school. Children with atypical development were excluded to ensure sample comparability and to avoid confounding effects, as their neurocognitive responses to physical activity interventions may differ substantially from typically developing peers. Interventions consisted of structured physical activity programs delivered in school or extracurricular settings, including aerobic exercise, motor skill training, cognitively engaging activities, or combined modalities. Studies were required to include a comparison condition, either an active control group, a passive control group, or an alternative intervention assessed at the same time points. Outcomes measured executive functions such as inhibitory control, working memory, and cognitive flexibility, or related cognitive processes assessed through standardized behavioral tasks, neurophysiological measures, or neuroimaging techniques. Eligible designs included randomized controlled trials, cluster-randomized trials, and crossover trials. Quasi-experimental and observational studies were excluded. The eligibility criteria applied in this review are summarized in Table 1.

**Table 1:** PICOS criteria for study eligibility.

Component	Description
Population (P)	Healthy children aged 6 to 12 years or attending primary school.
Intervention (I)	Structured physical activity programs conducted in school or extracurricular settings, including aerobic exercise, motor skill training, cognitively engaging activities, or combined modalities.
Comparison (C)	Active control group, passive control group, or alternative intervention assessed at the same time points.
Outcomes (O)	Executive functions (e.g., inhibitory control, working memory, cognitive flexibility) or related cognitive processes measured using standardized behavioral tasks, neurophysiological measures, or neuroimaging techniques.
Study Design (S)	Randomized controlled trials (RCTs), cluster-RCTs, and crossover trials. Quasi-experimental and observational designs were excluded.

EF = Executive Functions. Description of the Population, Intervention, Comparison, Outcomes, and Study Design criteria used to select studies included in this systematic review. Although crossover trials were eligible according to the PICOS criteria, no studies with this design were ultimately included after applying the eligibility and screening process.

Studies were excluded if they met any of the following criteria: review articles, book chapters, conference abstracts, or commentaries; insufficient information to calculate effect sizes after contacting the authors; non-target populations (for example, adolescents, preschool children, or clinical groups such as ADHD); or lack of a comparison group.

**Figure 1:**
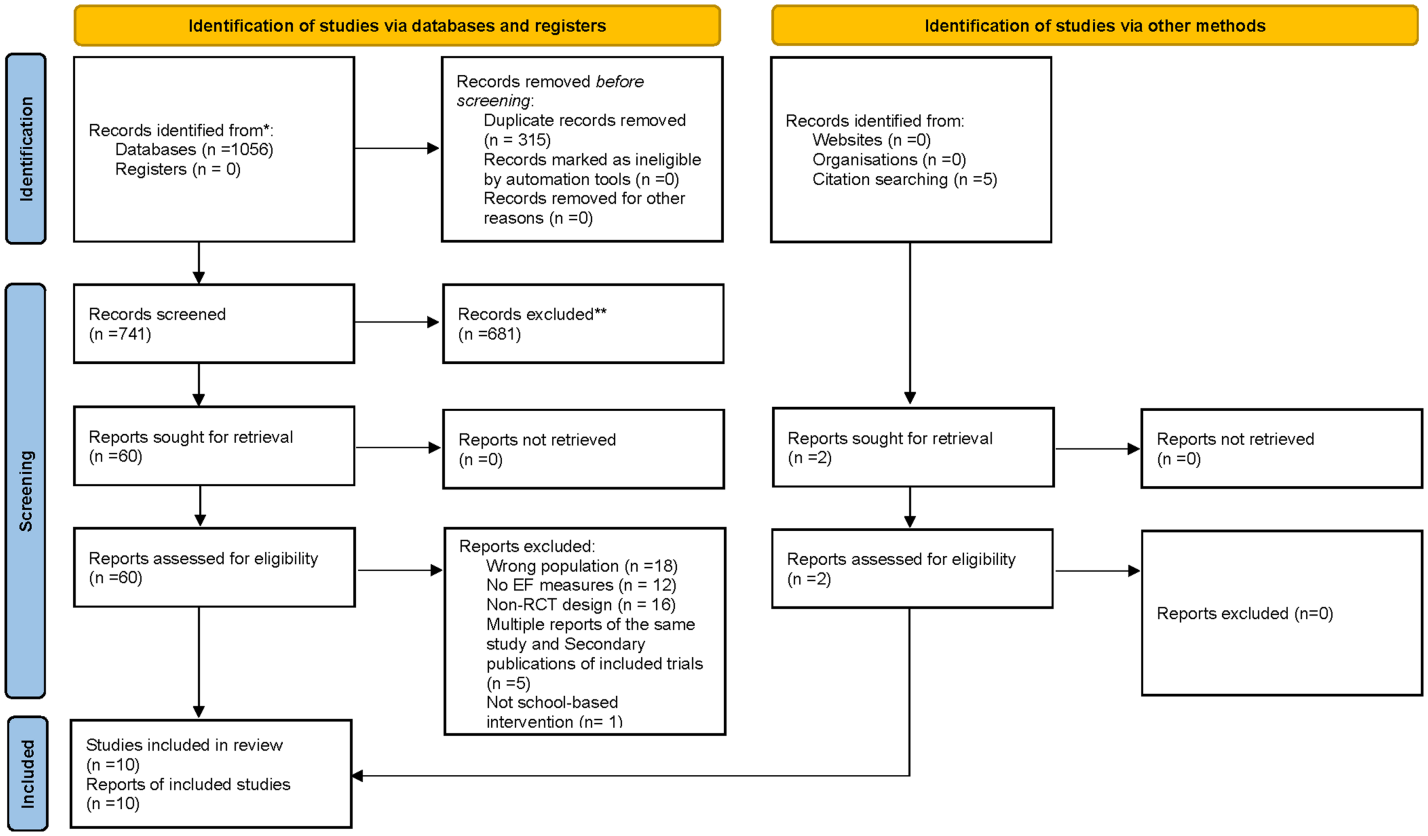
PRISMA 2020 flow diagram illustrating the process of study selection. Adapted from Page et al. (2021). The PRISMA 2020 statement an updated guideline for reporting systematic reviews. BMJ 372:n71.

The screening process was conducted in three phases. In the first phase, titles and abstracts were examined to remove duplicates and irrelevant records. In the second phase, abstracts were reviewed for eligibility based on the inclusion criteria. In the final phase, full-text articles were assessed in detail. Two independent reviewers carried out the selection process, and any discrepancies were resolved through discussion or consultation with a third reviewer until consensus was reached. The study selection process is illustrated in Figure 1.

One study (Kao et al., 2023) was excluded during full-text review upon confirmation that the intervention was conducted in a laboratory setting and not within a school or extracurricular environment, thus not meeting the inclusion criteria.

### 2.2 Data extraction

Data extracted from each eligible study included publication year, participant characteristics, study design, measurement instruments, executive function variables assessed, intervention details, and study settings. This information is presented in Table 2.

**Table 2:** Characteristics of the included studies.

Citation	Design	Sample (n)	Age	Duration	EF Variables	Main Instruments	Intervention	Frequency	Intensity	Time	Type	School setting/domain
Chatzopoulos et al. (2023)	RCT	67	~10.4 years	Single session	Inhibition, Working Memory, Flexibility	Flanker, Digit Span, Tower of London	Dance Active Break	1 session	Moderate-to-vigorous	5 min	Dance break	Classroom active break
Koutsandréou et al. (2016)	RCT	71	~9.4 years	10 weeks	Working Memory	Letter Digit Span	Motor Exercise	3/week	Moderate	30 min/session	Motor coordination training	PE lessons
Kvalø et al. (2017)	RCT	449	~10 years	10 months	Inhibition, Cognitive Flexibility, Working Memory	Stroop, Verbal Fluency, Digit Span, TMT	School PA Program	5/week	Moderate-to-vigorous	60 min/day	General physical activity	Curricular PE
Mazzoli et al. (2021)	RCT	141	6–8 years	6 weeks	Inhibition	Go/No-Go, fNIRS	Active Breaks (cognitive load)	3/week	Moderate	5–10 min/session	Cognitive active breaks	Classroom breaks
Oppici et al. (2020)	RCT	80	8–10 years	7 weeks	Working Memory	NIH List Sorting	Dance (cognitive load)	2/week	Moderate	45 min/session	Dance with cognitive demand	PE curriculum
Pesce et al. (2016)	Cluster RCT	460	5–10 years	6 months	Inhibition	Random Number Generation	Enriched PE	2/week	Moderate	60 min/session	PE with cognitive tasks	PE
Schmidt et al. (2015)	Controlled	181	10–12 years	6 weeks	Cognitive Flexibility	Flanker, N-Back	Team Games	2/week	Moderate-to-vigorous	45 min/session	Team games	PE or extracurricular
van den Berg et al. (2019a)	Cluster RCT	312	~10 years	5 weeks	Working Memory	Multiplication Test	Juggling & Math Lessons	4/week	Light to moderate	5–8 min/session	Physically active learning (jugg.)	Math curriculum (PAL)
van den Berg et al. (2019b)	Cluster RCT	512	9–12 years	9 weeks	Inhibition, Working Memory, Attention	Stroop, d2, ANT, Verbal Fluency	Just Dance Breaks	5/week	Moderate-to-vigorous	10 min/session	Dance with coordination	Classroom active breaks
Zhong et al. (2024)	RCT	80	7–12 years	12 weeks	Working Memory	N-back, fNIRS	Volleyball Training	3/week	Moderate (60–69% HRmax)	60 min/session	Team sport (open-skill)	Extracurricular

This table summarizes the 10 studies included in the final synthesis of the systematic review. Two studies by van den Berg et al. (2019a,b) are distinct investigations conducted by the same first author in the same year: one evaluated integrating juggling with math lessons, and the other assessed Just Dance active breaks.

PA = Physical Activity; PE = Physical Education; MVPA = Moderate to Vigorous Physical Activity; DL-PFC = Dorsolateral Prefrontal Cortex; PAL = Physically Active Learning; EF = Executive Functions; RCT = Randomized Controlled Trial; fNIRS = Functional Near-Infrared Spectroscopy; HRmax = Maximum Heart Rate; TMT = Trail Making Test; ANT = Attention Network Test.

Quantitative data was organized by executive function domain. Effect sizes reported for the same cognitive outcome were pooled to calculate aggregated estimates. When multiple outcomes were available within a single study, two criteria guided selection. For assessments providing several results, the outcome reflecting the highest cognitive demand was extracted (Álvarez-Bueno et al., 2017; Xue et al., 2019). For tasks reporting both accuracy and reaction time, accuracy was used as the primary indicator of cognitive performance. For example, in studies using the Flanker task to evaluate inhibitory control, the accuracy on incongruent trials was selected for synthesis.

Due to heterogeneity in study designs, intervention protocols, and measurement instruments, no quantitative meta-analysis was performed. Findings were synthesized narratively and organized by executive function domain, intervention characteristics, and methodological quality.

### 2.3 Risk of bias assessment

The risk of bias and methodological quality of the included studies were evaluated using a standardized checklist based on criteria commonly applied in exercise and cognitive intervention trials. The assessment considered eligibility criteria, random allocation, concealed allocation, blinding of participants, therapists, and assessors, retention rates, use of intention-to-treat analysis, and reporting of point estimates and variability measures. All evaluations were independently verified by a second reviewer to ensure consistency and accuracy. Interrater agreement was assessed using Cohen’s kappa coefficient, with k values >0.80 indicating strong agreement. Discrepancies were resolved through discussion or by consulting a third reviewer.

To facilitate transparency, Table 3 shows how the customized checklist domains align with the standardized ROB-2 tool. This adapted approach was informed by previous applications in exercise-cognition studies and aimed to capture contextual challenges typical of school-based interventions. Notably, our checklist diverges from RoB-2 in domains such as participant and assessor blinding, which are rarely feasible in educational PA trials. For instance, while RoB-2 requires strict blinding procedures, our tool acknowledges that blinding participants or teachers is often impossible in school-based interventions. Similar adaptations have been proposed in other reviews focused on complex behavioral trials in educational contexts.

**Table 3:** Comparison between the customized checklist and ROB-2 domains.

Customized checklist domain	Corresponding ROB-2 domain	Explanation
Random allocation	Bias arising from the randomization process	Whether the allocation sequence was truly random.
Concealed allocation	Bias arising from the randomization process	Whether allocation was concealed until participants were enrolled.
Blinding of participants	Bias due to deviations from intended interventions	Whether participants were blinded to group assignment to prevent expectation effects.
Blinding of outcome assessors	Bias in measurement of the outcome	Whether those assessing outcomes were unaware of group allocation.
Retention rate (>85%)	Bias due to missing outcome data	Whether sufficient data were retained to avoid attrition bias.
Intention-to-treat analysis	Bias due to deviations from intended interventions	Whether data analysis included all randomized participants regardless of adherence.
Between-group comparisons and reporting of variability	Bias in selection of the reported result	Whether results were reported transparently with measures of variability (e.g., SD, CI).

This table summarizes the correspondence between the customized risk of bias checklist used in this review and the domains assessed by the Cochrane ROB-2 tool. The checklist was designed to capture key methodological aspects relevant to school-based physical activity interventions targeting executive function outcomes.

Given the diversity of intervention formats and the focus on behavioral and educational outcomes, a customized checklist adapted from criteria commonly used in exercise-cognition trials was employed instead of the ROB-2 tool. This approach was intended to reflect specific features of school-based interventions. However, future reviews could complement this assessment with ROB-2 ratings to facilitate comparison with other systematic reviews. Additionally, given the low feasibility of blinding participants in school-based exercise protocols, a sensitivity analysis excluding the blinding domain may be considered in future applications of this checklist to offer an alternative perspective on study quality.

The results of the detailed quality assessment are summarized in Table 4, while the overall risk of bias ratings are presented in Table 5.

**Table 4:** Quality assessment of included studies.

Study	Eligibility criteria	Random allocation	Concealed allocation	Baseline similarity	Blinding subjects	Blinding therapists	Blinding assessors	Retention >85%	Intention to treat	Between-group comparison	Point & variability measures	Total score
Chatzopoulos et al. (2023)	Yes	Yes	Unclear	Yes	No	No	Unclear	Yes	No	Yes	Yes	**7**
Koutsandréou et al. (2016)	Yes	Yes	Unclear	Yes	No	No	Unclear	Yes	Unclear	Yes	Yes	**7**
Kvalø et al. (2017)	Yes	Yes	Unclear	Yes	No	No	Unclear	Yes	Yes	Yes	Yes	**8**
Mazzoli et al. (2021)	Yes	Yes	Yes	Yes	No	No	Yes	Yes	Yes	Yes	Yes	**10**
Oppici et al. (2020)	Yes	Yes	Unclear	Yes	No	No	Unclear	Yes	Yes	Yes	Yes	**8**
Pesce et al. (2016)	Yes	Yes	Unclear	Yes	No	No	Unclear	Yes	No	Yes	Yes	**7**
Schmidt et al. (2015)	Yes	Unclear	Unclear	Yes	No	No	Unclear	Yes	No	Yes	Yes	**6**
van den Berg et al. (2019a)	Yes	Yes	Unclear	Yes	No	No	Unclear	Yes	Yes	Yes	Yes	**8**
van den Berg et al. (2019b)	Yes	Yes	Unclear	Yes	No	No	Unclear	Yes	Yes	Yes	Yes	**8**
Zhong et al. (2024)	Yes	Yes	Yes	Yes	No	No	Yes	Yes	Yes	Yes	Yes	**10**

Quality assessment based on established criteria including randomization procedures, allocation concealment, blinding practices, participant retention, intention-to-treat analysis, and reporting of statistical measures. No crossover trials were included in the final sample, although they were eligible according to the PICOS criteria. Bold values indicate items judged as having a high risk of bias or serious methodological concern within the corresponding domain of the quality assessment tool. These values are highlighted to draw attention to potential limitations that could affect the reliability of the study findings.

**Table 5:** Risk of bias assessment.

Study	Random allocation	Concealed allocation	Blinding subjects	Blinding assessors	Intention to treat	Overall risk of bias
Chatzopoulos et al. (2023)	Yes	Unclear	No	Unclear	No	Some concerns
Koutsandréou et al. (2016)	Yes	Unclear	No	Unclear	No	Moderate risk
Kvalø et al. (2017)	Yes	Unclear	No	Unclear	Yes	Some concerns
Mazzoli et al. (2021)	Yes	Yes	No	Yes	Yes	Low risk
Oppici et al. (2020)	Yes	Unclear	No	Unclear	Yes	Some concerns
Pesce et al. (2016)	Yes	Unclear	No	Unclear	No	Moderate risk
Schmidt et al. (2015)	Unclear	Unclear	No	Unclear	No	Moderate risk
van den Berg et al. (2019a)	Yes	Unclear	No	Unclear	Yes	Some concerns
van den Berg et al. (2019b)	Yes	Unclear	No	Unclear	Yes	Some concerns
Zhong et al. (2024)	Yes	Yes	No	Yes	Yes	Low risk

Risk of bias was assessed using core methodological domains derived from the Cochrane RoB-2 tool. In addition, a customized checklist adapted to the specific challenges of school-based physical activity trials was applied (see Table 4; Supplementary Table 1). The overall risk categories reflect the number and severity of “No” or “Unclear” ratings across key domains. For clarity, “Intention to Treat” has been uniformly coded using “Yes” or “No”.

Given the frequent impossibility of blinding participants or teachers in school-based exercise interventions, we conducted a sensitivity analysis excluding the three blinding-related items from the quality checklist (participants, therapists, and assessors). When removing these domains, 9 out of the 10 included studies would be classified as low risk of bias based on adjusted total scores, suggesting that concerns about blinding may overestimate the methodological limitations of otherwise rigorous trials in this context. Only one study (Schmidt et al., 2015) remained at moderate risk due to unclear allocation procedures and absence of intention-to-treat analysis. The adjusted scores and reclassified risk levels are presented in Supplementary Table 1.

### 2.4 Data synthesis

Data were synthesized narratively due to substantial clinical and methodological heterogeneity across studies. This decision was based on marked variability in (1) intervention duration (acute vs. chronic), (2) intensity, frequency, and type of physical activity, (3) delivery mode and school setting (e.g., physical education, recess, classroom-based), and (4) outcome measures used to assess executive functions, including behavioral tasks, neuropsychological assessments, and neuroimaging techniques. Given these differences, a quantitative meta-analysis and estimation of statistical heterogeneity (e.g., I^2^) were not appropriate.

## 3 Results

### 3.1 Overview of included studies

A total of ten studies met the eligibility criteria and were included in the review. The interventions showed substantial variability in design, duration, and intensity, ranging from single-session activities to structured programs extending over several weeks or months. In most cases, the outcomes focused on executive functions and were assessed using standardized cognitive tasks. However, due to considerable clinical and methodological heterogeneity, particularly regarding intervention length, intensity, and the tools used to measure executive functioning, a formal estimation of statistical heterogeneity (e.g., I^2^) was not conducted.

The structure and duration of the interventions also varied widely. Some involved a single session lasting between 5 and 40 min, while others consisted of multi-week protocols ranging from four to twelve weeks. Session frequency and total dosage differed across studies.

Most interventions were delivered in school settings, including classrooms, gymnasiums, or recess periods, offering natural integration into the school day. A smaller number took place in structured after-school programs. The activities included aerobic exercise, motor skill development, dance-based routines, and cognitively engaging physical tasks designed to elicit executive function demands.

The executive function outcomes assessed included the three core components specified in the search strategy: inhibitory control, working memory, and cognitive flexibility. These domains were selected *a priori* as primary outcomes in line with the operational definition guiding the review. Some studies also reported secondary measures such as attention and processing speed, but only data corresponding to the predefined executive components were extracted for synthesis. Standardized tasks like the Flanker Task, Stroop Test, Trail Making Test, and N-back paradigms were commonly employed. Several studies also incorporated computerized testing protocols and neurophysiological techniques, including electroencephalography (EEG) and functional near-infrared spectroscopy (fNIRS).

### 3.2 Effects on executive functions

#### 3.2.1 Inhibitory control

Seven of the ten included studies assessed inhibitory control as a primary or clearly defined outcome: Chatzopoulos et al. (2023), Mazzoli et al. (2021), Schmidt et al. (2015), Pesce et al. (2016), Oppici et al. (2020), van den Berg et al. (2019a), and Kao et al. (2023). This function was typically evaluated through tasks such as the Flanker, Stroop, or Go/No-Go paradigms, which require participants to suppress automatic responses and manage conflicting stimuli under time constraints (Fan et al., 2002).

Two studies employed acute interventions and reported immediate, though modest, improvements. Chatzopoulos et al. (2023) observed enhanced performance on the Flanker task following a five-minute dance-based active break focused on rapid cue-response adaptation. Mazzoli et al. (2021) also found gains in a Stroop-like task after a single session of cognitively enriched classroom movement. In addition to behavioral improvements, their use of fNIRS revealed more efficient prefrontal activation, indicating neural optimization in inhibition-related processes.

Among chronic interventions, findings were more varied but generally favorable when cognitive challenge was present. Schmidt et al. (2015) documented significant gains in inhibitory control after a six-week program involving team games that demanded quick decision-making and strategy shifts. Pesce et al. (2016) reported similar improvements in children exposed to an enriched PE curriculum based on varied motor tasks and deliberate play. These interventions stand out for integrating unpredictability and executive challenge rather than relying solely on physical repetition.

Oppici et al. (2020) compared two dance conditions differing in cognitive load. Only participants in the high-load condition, who had to memorize, adapt, and coordinate complex routines, showed significant improvements in inhibition. In contrast, van den Berg et al. (2019a) found no effects after a nine-week dance program emphasizing imitation and repetition, with little novelty or demand for cognitive control. Finally, Kao et al. (2023), using a neurocognitive approach, found no meaningful behavioral or electrophysiological changes after a single bout of intense aerobic exercise, despite measuring both Stroop task accuracy and P3 amplitude.

The clearest benefits in inhibitory control emerged when physical activity was paired with sustained cognitive engagement, particularly in contexts that required suppressing dominant responses, adapting to changing rules, or maintaining attention under pressure. These results support theoretical models that highlight the role of novelty, complexity, and strategic demand in activating prefrontal circuits linked to self-regulation (Best, 2010; Diamond and Ling, 2016). They also align with the broader pattern observed in this review: interventions that challenge both body and mind tend to yield the strongest executive outcomes.

#### 3.2.2 Working memory

Working memory was assessed in seven of the included studies: Chatzopoulos et al. (2023), Mazzoli et al. (2021), Schmidt et al. (2015), Koutsandréou et al. (2016), Pesce et al. (2016), Oppici et al. (2020), and Zhong et al. (2024). Most used tasks like Digit Span, N-back, or recall-based measures to evaluate children’s ability to hold and manipulate information in real time.

Short interventions showed little effect. Chatzopoulos et al. (2023) tested a five-minute dance break and found no changes in performance. Mazzoli et al. (2021), using a similar approach with cognitively enriched activities, also failed to detect post-session improvements. In both cases, the sessions may have been too brief or not sufficiently demanding to influence memory systems that require repeated stimulation and consolidation over time.

The picture was different for longer interventions. Schmidt et al. (2015) ran a six-week program centered on team games involving strategy, shifting roles, and fast decision-making. Children showed higher accuracy on the N-back task, pointing to better working memory under pressure. Koutsandréou et al. (2016) trained children for ten weeks in either cardiovascular or motor coordination exercises. Both groups improved, but those in the coordination condition, focusing on balance, sequencing, and complex movement, did better, suggesting that physical complexity adds cognitive value.

Pesce et al. (2016) approached the issue through deliberate play. Their sessions combined motor tasks with embedded cognitive demands like rule adaptation and planning. The result: clear gains in working memory, likely driven by the dynamic and engaging nature of the program.

Zhong et al. (2024) added further depth with both behavioral and neural data. After twelve weeks of volleyball training, children responded faster and more accurately on N-back tasks. fNIRS data showed increased activation in the dorsolateral prefrontal cortex, supporting the idea that open skill sports, those requiring constant adjustment and anticipation, can reinforce working memory both functionally and structurally.

Not all programs worked. Oppici et al. (2020), for example, did not find significant changes in this domain. Their dance-based intervention focused more on inhibition and memory for movement sequences. The cognitive load may not have matched the specific demands of working memory tasks, which require active updating rather than simple recall.

The clearest benefits came from programs that lasted several weeks and combined physical effort with cognitive or coordinative challenge. When children had to adapt, plan, or react under changing conditions, memory performance improved. In contrast, interventions based on repetition, low variation, or short duration showed little impact. This is consistent with the idea that working memory responds best to activities that require constant monitoring, switching, and the flexible use of information (Best, 2010; Diamond and Ling, 2016).

#### 3.2.3 Cognitive flexibility

Five studies assessed cognitive flexibility using tasks involving rule shifting, task switching, or mental set adaptation: Schmidt et al. (2015), Oppici et al. (2020), van den Berg et al. (2019a), Mazzoli et al. (2021), and Chatzopoulos et al. (2023). In some cases, flexibility was not the primary focus, and the tools used did not clearly distinguish it from inhibition or attention.

Acute interventions showed limited effects. Chatzopoulos et al. (2023) tested a five-minute dance-based active break and found no improvement in task-switching performance. Similarly, Mazzoli et al. (2021), despite using cognitively enriched breaks and observing improvements in inhibition and prefrontal activation (via fNIRS), reported no measurable gains in flexibility. These findings suggest that short, isolated sessions, especially those with limited cognitive variability, may be insufficient to elicit change in this domain.

More promising results were observed in longer programs. Schmidt et al. (2015) implemented a six-week team game intervention involving shifting rules, rotating roles, and time-constrained decisions. Children improved on mental flexibility tasks, indicating that repeated exposure to dynamic, cognitively demanding contexts can foster development in this area. Oppici et al. (2020) found a similar effect: only participants in the high cognitive load group improved in task-switching performance, despite engaging in the same motor activity as the control group. This points to the decisive role of cognitive complexity in eliciting gains.

In contrast, van den Berg et al. (2019a) found no improvement after a nine-week Just Dance intervention. Although physically active and sustained, the program relied on imitation and repetition, offering little strategic or cognitive challenge.

Taken together, these findings suggest that flexibility improves primarily when activities require children to adapt to changing conditions and switch between strategies under pressure. Programs based solely on repetition, even if physically demanding, rarely produce measurable effects. Moreover, the sensitivity of the assessment tools used may influence outcomes. Some studies relied on instruments that may not detect subtle gains in executive adaptability, which could partly explain the inconsistent results. Overall, cognitive flexibility appears to require both sustained engagement and task novelty, as well as precise measurement to register change.

#### 3.2.4 Attention and processing speed

Attention and processing speed were assessed in five of the ten included studies: Chatzopoulos et al. (2023), Mazzoli et al. (2021), Schmidt et al. (2015), Pesce et al. (2016), and Zhong et al. (2024). These constructs were typically measured as secondary outcomes, using tools such as the d2 Test of Attention, the Stroop Test, or reaction time tasks embedded within executive paradigms like the Flanker or Go/No-Go (Fan et al., 2002).

Short-term interventions yielded limited effects. Chatzopoulos et al. (2023) observed no changes in attentional performance or reaction time following a five-minute dance-based active break. Similarly, Mazzoli et al. (2021), despite reporting gains in inhibitory control and increased prefrontal activation via fNIRS, found no improvements in these domains. These findings align with previous meta-analyses suggesting that attentional improvements are unlikely to occur after isolated sessions, even when cognitively enriched (Chang et al., 2012; Best, 2010).

More favorable results were observed in longer interventions. Schmidt et al. (2015) reported improvements in selective attention after 6 weeks of structured team games designed to combine movement with rule adaptation and decision-making. Pesce et al. (2016) found similar benefits using a curriculum based on varied motor challenges and deliberate play. In both cases, attentional gains appeared to stem from sustained cognitive involvement rather than physical exertion alone.

Additional support comes from Zhong et al. (2024), who reported faster reaction times and increased prefrontal activation following a twelve-week volleyball program involving anticipatory decision-making and dynamic motor planning. Although attention was not isolated as a primary outcome, the findings suggest greater processing efficiency in task execution. Other relevant evidence, though excluded from the main synthesis due to design differences, includes Kvalø et al. (2017), who observed small improvements in attentional control after ten months of daily school-based activity, and van den Berg et al. (2019b), who reported reduced reaction times following a dual-task intervention combining juggling with academic instruction.

In light of these findings, attention and processing speed appear to benefit from school-based physical activity primarily when interventions are sustained and cognitively demanding. However, the magnitude of these effects was generally smaller and less consistent than those observed for inhibitory control or working memory. This pattern likely reflects the secondary emphasis of these domains in many intervention designs, the short duration of some programs, and the use of non-specific or low-sensitivity instruments. As Dixon et al. (2025) note, variability in outcome measurement continues to limit cross-study comparability and interpretability. Future research should treat attention and speed as primary outcomes, using validated and ecologically relevant tools, particularly in school contexts where these skills are critical for academic functioning.

### 3.3 Methodological quality and risk of bias

The methodological quality of the included studies varied considerably. As summarized in Table 4, all trials reported clear eligibility criteria and applied random allocation procedures, either individually or at the cluster level. While most studies did not describe specific methods for allocation concealment, a few, including Mazzoli et al. (2021) and Zhong et al. (2024), reported adequate procedures for maintaining allocation concealment during randomization.

Blinding of participants and therapists was consistently absent, which is common in exercise interventions. Only Zhong et al. (2024), reported blinding of outcome assessors, typically when neurophysiological measures like EEG or fNIRS were used.

Retention rates were generally high, with most trials exceeding 85% participant retention. Analyses using the intention-to-treat principle were explicitly described in approximately half of the studies. Nearly all trials provided between-group comparisons along with measures of central tendency and variability.

Based on the predefined criteria, two studies were classified as having a low overall risk of bias. The remaining trials were considered to present a moderate risk of bias, mainly due to the lack of concealed allocation and the absence of blinding procedures.

### 3.4 Overall synthesis of findings

The results of this review suggest that structured physical activity interventions can contribute to improvements in executive functions among children aged 6 to 12 years. Acute exercise sessions, especially those with moderate-to-vigorous intensity, often produced short-term gains in inhibitory control. Some studies also reported positive changes in attention and processing speed, though these effects tended to be modest and varied across interventions. Evidence for improvements in working memory and cognitive flexibility after single exercise bouts was limited.

Longer interventions delivered over several weeks showed clearer benefits across executive function domains. Programs that included cognitively engaging elements, such as team games with decision-making or enriched physical education, were more consistently linked to gains in working memory and flexibility. Interventions focused only on aerobic or motor skill practice also demonstrated benefits in some cases, although results were not uniform.

The methodological quality of the included trials was generally moderate. Most studies applied random allocation and achieved high participant retention, but details about allocation concealment and blinding were often missing. These limitations should be considered when interpreting the size and reliability of the reported effects.

The findings support the potential of structured physical activity, particularly approaches that combine movement with cognitive challenges, as a strategy to strengthen executive functions in school-aged children. Further research using rigorous trial designs and standardized assessment methods would help clarify which intervention characteristics are most effective. Table 6 provides a summary of the number of studies reporting positive or non-significant effects across executive function domains and intervention types.

**Table 6:** Summary of effects by executive function domain and intervention type.

Executive function domain	Intervention type	Studies with positive effect (*n*)	Studies with no significant effect (*n*)
Inhibitory Control	Acute	2	0
Inhibitory Control	Chronic	4	2
Working Memory	Acute	0	3
Working Memory	Chronic	5	1
Cognitive Flexibility	Acute	0	1
Cognitive Flexibility	Chronic	3	3

This table summarizes the number of included studies reporting significant improvements or no significant effects in each executive function domain, according to whether the intervention was acute (single session) or chronic (delivered over multiple weeks).

Shaded cells indicate combinations with the strongest overall evidence of positive effects across studies.

## 4 Discussion

### 4.1 Summary of main findings

This systematic review examined the effects of structured physical activity interventions on executive function (EF) development in children aged 6 to 12. The evidence confirms that physical activity can support cognitive development in this age group, but the strength and reliability of these effects vary depending on the EF domain, the characteristics of the intervention, and the cognitive demands embedded in the activities.

Inhibitory control was the most responsive executive domain across the reviewed studies. Improvements were observed not only in long-term programs but also in acute interventions, provided that tasks involved rapid cue adaptation, response inhibition, or rule changes. For instance, Chatzopoulos et al. (2023) and Mazzoli et al. (2021) reported significant gains following brief, cognitively engaging activity breaks. Chronic programs such as those by Schmidt et al. (2015), Pesce et al. (2016), and Oppici et al. (2020) yielded even stronger effects when the activities required strategic decision-making and shifting between roles or rules. In contrast, van den Berg et al. (2019a), which relied on repetitive dance routines with limited novelty, found no improvements in inhibition, highlighting the importance of cognitive engagement.

Working memory showed more variable but generally positive results. Stronger effects were associated with interventions of longer duration that combined physical exertion with coordinative or strategic complexity. For example, Schmidt et al. (2015), Pesce et al. (2016), and Zhong et al. (2024) all implemented activities that required children to retain, update, and manipulate information under changing conditions. In contrast, studies focusing on less cognitively demanding movement, such as Koutsandréou et al. (2016) or Kao et al. (2023), reported more modest or inconsistent outcomes, suggesting that physical intensity alone is insufficient to improve working memory performance.

Cognitive flexibility proved more resistant to change. Only a few studies targeted this domain directly, and their results were mixed. Oppici et al. (2020) found that only the high-cognitive-load group in their dance-based intervention showed improvement in task switching. Mazzoli et al. (2021) and Chatzopoulos et al. (2023) observed no measurable effects after single-session breaks, even though both incorporated cognitively engaging tasks. These findings suggest that flexibility may require sustained exposure to unpredictable or strategically variable contexts, conditions that were not consistently present in the interventions reviewed.

Attention and processing speed were assessed in several studies as secondary outcomes (e.g., Schmidt et al., 2015; Mazzoli et al., 2021; Zhong et al., 2024). Some programs reported moderate gains, particularly those involving mental engagement in dynamic settings, yet the overall pattern was less robust. Variability in instruments and outcome definitions may explain some of these inconsistencies.

The pattern that emerges across studies is that programs combining physical activity with cognitive challenge, novelty, and decision-making outperform those based purely on repetition or aerobic intensity. Activities designed with embedded executive demands, such as rule shifting, unpredictability, and strategic adaptation, consistently yielded better results. This aligns with prior theoretical models (Diamond and Ling, 2016; Best, 2010), while offering a more nuanced understanding of which functions improve and under what conditions.

Recent trials also expanded the methodological landscape by incorporating neurophysiological measures. Mazzoli et al. (2021) and Zhong et al. (2024) used functional near-infrared spectroscopy (fNIRS), while Kao et al. (2023) employed event-related potentials (ERP) to assess neural markers of executive processing. These studies suggest that prefrontal activation patterns are sensitive not only to exercise intensity but also to the cognitive demands and novelty of the task, reinforcing the role of mental engagement as a key mechanism of change.

By identifying how different EF domains respond to specific types of interventions, and by incorporating both behavioral and neurophysiological data, this review contributes a more differentiated and actionable perspective on the potential of school-based physical activity programs to support children’s cognitive development.

### 4.2 Interpretation by domain

#### 4.2.1 Inhibitory control

Of the three executive function domains analyzed, inhibitory control was the most consistently responsive to physical activity interventions. Gains were observed in both acute and chronic formats, though their strength depended on the cognitive demands, structure, and duration of the intervention.

Acute interventions, even those lasting just a few minutes, demonstrated measurable improvements when they incorporated elements of cognitive engagement. Chatzopoulos et al. (2023) reported that a five-minute dance-based active break improved children’s performance on the Flanker task, likely due to the rapid cue adaptation and coordinated response requirements embedded in the activity. Similarly, Mazzoli et al. (2021) found enhanced inhibitory control following classroom-based breaks involving movement synchronized with attentional cues and rhythmic variation. Notably, this study also documented more efficient activation of the prefrontal cortex using fNIRS, suggesting that cognitive-physical integration, even briefly applied, can produce functional neural changes related to inhibition.

Chronic programs yielded broader, and often more durable, improvements, particularly when they included elements of decision-making, rule variability, or strategic adaptation. Schmidt et al. (2015), for example, designed a six-week team game program where children regularly faced shifting roles and dynamic rule changes. This group outperformed peers in a traditional PE control condition on the Stroop test, indicating that structured cognitive engagement during play was a central mechanism of improvement.

Pesce et al. (2016) adopted a curriculum grounded in deliberate play, encouraging self-regulation, exploration, and variability. Children exposed to this approach showed significant post-intervention gains in inhibitory control, highlighting the role of autonomy and adaptable task structure in fostering executive development. Oppici et al. (2020) reached similar conclusions: their seven-week dance-based intervention led to improvements only in the group exposed to high cognitive load, where participants were challenged to memorize complex sequences, maintain temporal coordination, and attend to multiple cues simultaneously. The contrast with the low-load group supports a dose-response effect between cognitive demand and inhibitory gains.

Not all interventions were effective. Van den Berg et al. (2019a) implemented a nine-week Just Dance routine during classroom time but reported no significant improvement in inhibitory performance. The repetitive and imitation-based nature of the activity likely failed to activate the executive systems required for suppression and adaptation. Likewise, Kao et al. (2023) assessed Go/No-Go and Flanker performance following a single session of high-intensity aerobic or interval exercise and found no post-test gains, even when measured with ERP markers. Their findings suggest that intensity alone, without embedded cognitive challenge, may be insufficient to trigger improvements in inhibition.

Some studies offered indirect or mixed evidence. Koutsandréou et al. (2016), although focused primarily on working memory, reported better response control after a motor coordination program, though inhibition was not directly measured. Zhong et al. (2024), whose volleyball training intervention involved anticipation and tactical decisions, observed generalized executive improvements, but did not isolate inhibitory control as a specific outcome.

Altogether, the most effective approaches were those that combined physical intensity with clear executive demands, particularly those involving real-time decision-making, interference control, and adaptation to dynamic task rules. These findings refine and extend earlier theoretical proposals (Diamond and Ling, 2016), showing that inhibition benefits not merely from movement, but from structured environments that challenge children’s ability to filter distractions and regulate responses. The neural data from Mazzoli et al. (2021) further suggest that these behavioral effects may reflect increased neural efficiency in prefrontal regions involved in self-regulation.

#### 4.2.2 Working memory

Compared to inhibitory control, the evidence regarding working memory was more heterogeneous, both in terms of outcomes and intervention characteristics. While several studies reported positive effects, particularly among longer programs with embedded cognitive and coordinative challenges, the findings were not consistent across all formats or designs.

Short, single-session interventions generally showed little or no effect on working memory. Chatzopoulos et al. (2023) found no improvement following a five-minute dance-based active break, despite its rhythmic and attentional demands. Similarly, Mazzoli et al. (2021) used brief classroom activities that combined movement with attentional cues yet observed no gains in computerized working memory tasks. These results suggest that isolated sessions, even when cognitively engaging, may not provide the sustained or cumulative stimulation necessary to enhance updating and manipulation processes. This interpretation is reinforced by the study of Kao et al. (2023), conducted in a controlled laboratory setting, which also reported null effects on working memory following a single bout of aerobic or interval exercise. Despite their methodological rigor and inclusion of neuroelectric measures, the absence of cognitive structuring in the physical tasks may have limited the potential for impact.

In contrast, chronic interventions produced more promising outcomes, particularly when combining physical effort with cognitive or coordinative demands. Schmidt et al. (2015) implemented a six-week program centered on team games requiring rapid decision-making, attentional shifts, and adaptation to changing rules. Children in the experimental group improved significantly on N-back tasks compared to controls, highlighting the role of dynamic, unpredictable environments in stimulating executive processes. Similarly, Koutsandréou et al. (2016) compared aerobic and coordination-based training over 10 weeks and found working memory benefits in both groups, with greater improvements in the coordination condition. This suggests that activities involving sequencing, bilateral integration, and balance, key components of coordinative training, are especially beneficial for the cognitive operations involved in working memory.

Pesce et al. (2016) also supported this view with a PE curriculum based on deliberate play. By incorporating variability, flexible rule structures, and self-regulated problem-solving tasks, the intervention promoted meaningful gains in working memory, presumably due to the high level of mental engagement and motivational appeal. Further support comes from Zhong et al. (2024), who added a neurophysiological dimension. After 12 weeks of volleyball training, children showed improved accuracy and speed in N-back performance, along with increased activation in the dorsolateral prefrontal cortex, a region central to working memory function. These results suggest that sustained exposure to open-skill physical activity, when cognitively and socially demanding, can induce measurable functional adaptations in executive control networks.

However, not all long-term programs were effective. Van den Berg et al. (2019a) conducted a nine-week Just Dance intervention during school hours but did not observe significant improvements in working memory. The program prioritized imitation and rhythm, with limited variation or active problem-solving, which may explain the lack of cognitive transfer. Likewise, Oppici et al. (2020) tested a dance-based PE curriculum with two levels of cognitive load, but neither version led to improvements in working memory tasks. Although the high-load group benefited in terms of inhibition, it seems that the nature of the choreography, focused on sequence reproduction rather than flexible adaptation, may not have sufficiently targeted working memory demands.

The heterogeneity in findings underscores the importance of moderating variables, such as intervention duration, type of motor task, cognitive load, and assessment sensitivity. Interventions that integrated task variability, anticipatory decision-making, or strategic planning were more likely to produce benefits than those relying on fixed routines or passive repetition. Similarly, outcome measures with low ecological validity or insufficient cognitive challenge may have failed to detect subtle changes, particularly when used in studies with limited intervention exposure.

These findings refine existing theoretical models (Best, 2010; Diamond and Ling, 2016) by identifying more precisely the kinds of intervention characteristics that support working memory development in school-aged children. Rather than aerobic load alone, it appears that the combination of motor coordination, cognitive engagement, and sustained exposure is what enables meaningful improvements. Although neural evidence remains limited to a few studies, results such as those from Zhong et al. (2024) suggest that well-designed programs may produce both behavioral and neurofunctional changes.

#### 4.2.3 Attention and processing speed

Only a few studies in this review assessed attention or processing speed as distinct outcomes, and most did so as secondary variables within broader executive function protocols. These domains were typically measured using the d2 Test of Attention, the Stroop Task, or reaction time assessments embedded in Flanker or Go/No-Go paradigms.

Short interventions generally produced limited and inconsistent effects. Chatzopoulos et al. (2023) conducted a five-minute dance-based active break but found no significant improvement in attentional performance or response speed. Mazzoli et al. (2021), using similarly brief cognitively enriched classroom sessions, observed increased prefrontal activation in regions associated with attention, but this neural efficiency was not reflected in behavioral outcomes. These findings suggest that short-duration sessions, even when cognitively engaging, may lack the cumulative load or sustained stimulation needed to generate measurable improvements in attention or processing speed.

More structured and extended programs yielded clearer evidence of change. Schmidt et al. (2015) reported gains in selective attention after a six-week intervention centered on team games requiring constant shifts in strategy and attentional control. Pesce et al. (2016) also observed attentional improvements following a physical education curriculum based on deliberate play, emphasizing adaptation to changing rules and complex motor responses. In both studies, attentional benefits appeared to stem more from the executive demands of the activities than from their aerobic intensity.

Kvalø et al. (2017) provided additional support for the role of sustained exposure. Their ten-month school-wide intervention led to small but positive effects on attentional control, even though the program lacked intensive cognitive elements. This suggests that routine integration of physical activity over time may have a cumulative effect on attentional networks, albeit weaker than that observed in cognitively enriched protocols.

Zhong et al. (2024) reported faster reaction times after a twelve-week volleyball training program, accompanied by increased activation in prefrontal regions measured through fNIRS. The open-skill nature of volleyball, with its rapid perceptual-motor demands and anticipatory requirements, likely contributed to improvements in processing efficiency. A similar effect was observed in van den Berg et al. (2019b), where children engaged in juggling integrated with math instruction. Although attention was not isolated as a distinct outcome, reduced reaction times suggested enhanced attentional switching and dual-task coordination.

Taken as a whole, these findings indicate that attention and processing speed may benefit from school-based physical activity programs when they are sustained over time and include cognitively demanding elements. Novelty, unpredictability, and the need for sustained focus seem to be more critical than physical effort alone. Although improvements in these domains were generally more modest than those seen in core executive functions, the evidence points to their sensitivity to well-designed interventions that integrate movement with mental challenge.

#### 4.2.4 Cognitive flexibility

Cognitive flexibility was the least systematically explored of the three core executive functions in the reviewed studies. Only a few trials addressed it explicitly, and in most cases, it was assessed in conjunction with broader executive outcomes rather than as an independent construct.

Acute interventions offered little evidence of efficacy. In Chatzopoulos et al. (2023), a five-minute dance-based break failed to yield improvements in task-switching performance. Similarly, Mazzoli et al. (2021) found no behavioral changes in flexibility-related tasks, despite observing more efficient prefrontal activation during short, cognitively enriched activities. These results suggest that brief exposures, even when engaging, may not provide the sustained cognitive stimulation required to elicit meaningful changes in flexible control processes.

By contrast, several chronic interventions showed more favorable outcomes. Schmidt et al. (2015) reported significant improvements following a six-week program involving dynamic team games with unpredictable rule changes and shifting roles. The demand for ongoing strategic adjustment likely activated mechanisms associated with cognitive flexibility. In a similar vein, Pesce et al. (2016) implemented a deliberately varied PE curriculum in which children had to continuously adapt to evolving motor and cognitive demands. Improvements observed in this context may reflect enhanced ability to shift between mental sets and action plans.

Some studies offered indirect indications of flexibility-related benefits. Zhong et al. (2024), although not targeting this domain explicitly, found enhanced performance in tasks requiring complex response selection and perceptual shifting. These behavioral gains were accompanied by increased activation in dorsolateral prefrontal regions, suggesting a broader enhancement in executive adaptability.

However, not all chronic programs produced clear effects. Oppici et al. (2020) tested a dance-based curriculum with varying cognitive load, but neither condition yielded measurable gains in flexibility. Similarly, van den Berg et al. (2019a) implemented a classroom-based Just Dance routine emphasizing imitation and repetition and reported no improvement. In both cases, the absence of strategic variability, decision-making, or unpredictable constraints may have limited the recruitment of flexible control mechanisms.

Overall, the evidence suggests that flexibility is less responsive to generic physical activity than to interventions that continuously challenge children to shift perspectives, strategies, or goals. Unlike inhibition, which can benefit from brief, well-designed tasks, or working memory, which may respond to sustained motor-cognitive demands, flexibility appears to require frequent exposure to novel, contextually rich experiences. The relatively small number of studies that addressed this domain directly, and the methodological variation in how it was assessed, highlight the need for future research that isolates flexibility more precisely and explores the specific features of activity design that promote its development.

### 4.3 Practical implications and applicability

The evidence reviewed highlights that school-based physical activity can contribute meaningfully to executive function development, provided that interventions are carefully designed. Short cognitively enriched movement breaks, such as rhythm-based activities requiring attentional switching or cue adaptation, can temporarily enhance inhibition and readiness to learn. Although their effects are transient, they are low-cost, easy to implement, and well-suited to daily classroom routines.

More durable improvements, especially in working memory and inhibitory control, require sustained interventions delivered multiple times per week over several weeks. Programs with explicit cognitive demands, such as variable rules, strategic decision-making, or coordination under uncertainty, were consistently more effective than those based solely on repetition, imitation, or aerobic effort. Physical activity alone is insufficient; the interaction between motor and cognitive demands appears essential to elicit executive benefits.

This review offers added precision by mapping specific executive domains to distinct task characteristics. For example, working memory gains were more likely when tasks involved sequencing and updating under pressure, while flexibility required frequent adaptation to changing goals or rules. These associations have rarely been described with such clarity in prior reviews and can inform the pedagogical design of PE sessions or extracurricular activities.

Effective programs prioritize cognitive quality over quantity of movement. Instead of increasing total PE time, schools may achieve better outcomes by integrating short, cognitively demanding tasks throughout the day or by enriching existing physical education with strategic challenges. These approaches are adaptable to varied educational settings but require adequate teacher training and curricular flexibility.

Viewing physical activity as an opportunity to train core cognitive processes shifts its role from ancillary to integral within educational planning. When embedded in broader approaches that emphasize metacognition, self-regulation, and play-based learning, it becomes a vehicle not only for physical health but for long-term cognitive development.

In addition to these practical recommendations, this review offers distinct contributions by analyzing executive function domains separately, emphasizing the role of cognitive load in shaping outcomes, and incorporating neurophysiological evidence from studies using fNIRS and EEG. These features provide a more precise framework for understanding how school-based physical activity can enhance cognitive development.

### 4.4 Limitations of the review

Several limitations of this review should be acknowledged. Although the search strategy was comprehensive and protocol-driven, it was restricted to publications in English, potentially introducing language bias and excluding relevant non-English studies.

Considerable heterogeneity was observed across interventions regarding duration, frequency, cognitive content, and assessment methods. This variability precluded meaningful meta-analysis and limited the comparability of results, particularly across executive function domains. While this heterogeneity reflects the diversity of school-based practices, it complicates the identification of specific program features responsible for cognitive effects.

Methodological quality across studies was generally moderate. Few trials reported allocation concealment or assessor blinding, increasing risk of bias. In addition, outcome measures for executive functions varied widely, often lacking standardization or sensitivity, which reduces confidence in between-study comparisons. A tailored risk-of-bias checklist was used to better reflect the specific challenges of educational interventions, although this limits direct comparisons with reviews using tools like ROB-2.

Most included studies were conducted in Western, high-income countries with relatively homogeneous samples. This restricts the generalizability of findings and overlooks sociocultural factors that may shape both engagement in physical activity and cognitive outcomes. Individual-level moderators such as age, baseline fitness, and cognitive profile were rarely explored systematically.

The inclusion of both acute and chronic interventions provided a broader picture of the field but also introduced interpretative complexity. Short-term benefits observed after single sessions cannot be assumed to mirror long-term developmental effects. Studies rarely followed up participants beyond the immediate post-intervention period, limiting conclusions about sustainability.

While risk of bias was assessed (see Tables 4, 5), publication bias was not formally analyzed due to the narrative synthesis and small number of comparable studies. Similarly, although the PROSPERO protocol included plans to apply the GRADE framework, the marked methodological and clinical variability across studies made formal certainty assessments unfeasible.

These limitations underscore the need for better harmonization in intervention protocols and outcome measures. More robust trials with diverse samples, standardized assessments, and longer follow-up periods are essential to refine our understanding of how, for whom, and under what conditions physical activity can foster executive function development in childhood.

### 4.5 Directions for future research

Despite growing interest in the cognitive benefits of school-based physical activity, the field continues to grapple with methodological inconsistencies and conceptual ambiguity. Moving forward will require more than replicating existing exercise protocols. It demands sharper models and clearer conditions under which movement contributes meaningfully to executive function development.

First, trial design must improve. Although randomized controlled trials are increasingly common, many still rely on small samples, unclear randomization, lack of blinding, or *post hoc* outcome switching. Future studies should commit to transparent preregistration, intention-to-treat analyses, and active control conditions that go beyond “business as usual.” Reporting on implementation fidelity, participant adherence, and contextual factors is essential if findings are to inform real-world practice.

Second, there’s a need to define the effective “dose” of intervention. Instead of isolating variables like frequency or duration, trials should examine how combinations of session length, intensity, and cognitive load affect different executive domains. Factorial designs or adaptive protocols could help establish both minimum thresholds and saturation points. This knowledge would allow schools to plan efficient, scalable programs that fit within existing curricular structures.

Third, individual variability deserves greater attention. Too many studies treat children as a uniform group, overlooking differences in development, fitness, and cognitive baseline. Future research should stratify samples by age, sex, socioeconomic status, and executive profile, and apply interaction models or machine learning to identify for whom interventions work best. Tailored approaches could transform the field from general recommendations to precision-oriented practice.

Fourth, integrating acute and chronic perspectives is critical. Most studies focus on one or the other, missing the chance to track how short-term gains evolve, or fade, over time. Trials with immediate, midterm, and follow-up assessments could shed light on cognitive trajectories, sustainability, and links to academic and behavioral outcomes. Longitudinal studies remain scarce and urgently needed.

Fifth, broader socio-cultural representation is essential. Most available evidence comes from Western, high-income countries with relatively homogeneous school settings. Expanding research into more diverse populations would clarify how cultural context, educational norms, and structural inequalities shape both implementation and outcomes. Involving teachers and students directly in program design could also improve ecological validity and relevance.

Finally, theoretical clarity must improve. Many studies rely on generic assumptions about exercise and cognition without articulating how movement influences specific brain systems. Stronger theoretical grounding, drawing from developmental neuroscience, embodied cognition, or ecological dynamics, can sharpen hypotheses and guide the use of neurophysiological tools like fNIRS, EEG, or dual-task paradigms.

In short, the key question is no longer whether physical activity can enhance executive function. The field must now ask: what kind of movement, under what conditions, for which children, and through which mechanisms? These priorities align with the differentiated effects observed in this review and highlight the need to move from global prescriptions toward more targeted, evidence-based applications of physical activity in educational settings.

## 5 Conclusion

This review examined how structured physical activity in school settings affects executive functions in children aged 6 to 12. The evidence shows that these interventions can enhance cognitive development, but only under specific conditions. Gains were most pronounced when physical activities embedded cognitive challenges, requiring planning, decision-making, rule shifting, or attentional control, rather than relying on repetition or pure aerobic effort.

Inhibitory control emerged as the most responsive domain, even in brief interventions, when tasks involved suppressing impulses or resolving interference. Working memory improvements were more selective and linked to programs that emphasized coordination, sequencing, or rule-based adaptation. Cognitive flexibility proved harder to influence, often requiring longer exposure and greater contextual variability to elicit change.

What sets this review apart is its differentiated analysis by executive function domain, its focus on cognitive task design rather than physical intensity alone, and the inclusion of recent studies using neurophysiological tools such as fNIRS and EEG. These studies point to changes not only in behavior but also in enhanced functional activation in executive brain regions, suggesting that well-structured physical activity may support neurocognitive development at multiple levels.

Programs that failed to produce benefits often shared common features: fixed routines, minimal decision-making, or a lack of novelty. This highlights a key insight: movement alone is not enough. The cognitive architecture of the activity determines its impact.

These findings offer a practical guide for schools: short cognitively enriched breaks can sharpen attention and inhibition, while longer, more complex interventions are needed to support sustained gains in working memory or flexibility. Rather than increasing physical activity time indiscriminately, educational efforts should focus on integrating cognitively demanding formats within existing structures.

Further progress in this field will depend on more rigorous and theory-driven research. Future studies should identify optimal combinations of frequency, duration, and cognitive load, explore individual variability in response, and include longitudinal follow-ups. A better understanding of how specific executive functions respond to different types of movement will make it possible to design targeted, scalable interventions that align with developmental and educational priorities.

This review contributes a more nuanced understanding of how, when, and why physical activity can enhance executive functioning in childhood. It moves beyond general claims by mapping the specific conditions that make cognitive benefits more likely, and more meaningful, in real school environments.

## Data Availability

The original contributions presented in the study are included in the article/Supplementary material, further inquiries can be directed to the corresponding author.
