# Supplementary material for: Effects of school-based physical activity programs on executive function development in children: a systematic review
Source: Front Psychol. 2025 Sep 3;16:1658101. doi: 10.3389/fpsyg.2025.1658101 (PMC12440757; doi:10.3389/fpsyg.2025.1658101)
Supplement: Supplementary file 1 [file Table_1.DOCX]

**Supplementary Table 1. Sensitivity analysis of study quality excluding blinding-related items**

| **Study** | **Original Score (out of 11)** | **Score Without Blinding (out of 8)** | **Adjusted % Score** | **Adjusted Risk of Bias** |
| --- | --- | --- | --- | --- |
| Chatzopoulos et al. (2023) | 7 | 7 | 87.5% | Low risk |
| Koutsandreou et al. (2016) | 7 | 7 | 87.5% | Low risk |
| Kvalø et al. (2017) | 8 | 8 | 100% | Low risk |
| Mazzoli et al. (2021) | 10 | 8 | 100% | Low risk |
| Oppici et al. (2020) | 8 | 8 | 100% | Low risk |
| Pesce et al. (2016) | 7 | 7 | 87.5% | Low risk |
| Schmidt et al. (2015) | 6 | 6 | 75.0% | Some concerns |
| van den Berg et al. (2019a) | 8 | 8 | 100% | Low risk |
| van den Berg et al. (2019b) | 8 | 8 | 100% | Low risk |
| Zhong et al. (2024) | 10 | 8 | 100% | Low risk |

*Note:* This table presents a sensitivity analysis of study quality, excluding the three blinding-related items (participants, therapists, and assessors), which reduces the maximum possible score from 11 to 8. Adjusted risk of bias classifications are based on the proportion of positive ratings relative to this new maximum.
